# Molecular and morphological identification of *Lernaea* spp. in Cyprinid fishes from two districts in Yogyakarta, Indonesia

**DOI:** 10.14202/vetworld.2023.851-857

**Published:** 2023-04-25

**Authors:** Joko Prastowo, Dwi Priyowidodo, Yudhi Ratna Nugraheni, Ana Sahara, Wisnu Nurcahyo, Vika Ichsania Ninditya

**Affiliations:** 1 Department of Parasitology, Faculty of Veterinary Medicine, Universitas Gadjah Mada, Yogyakarta, Indonesia; 2 Graduate Student of Veterinary Sciences, Faculty of Veterinary Medicine, Universitas Gadjah Mada, Yogyakarta, Indonesia

**Keywords:** *18S rDNA*, *28S rDNA*, aquaculture, *Lernaea cyprinacea*, parasite

## Abstract

**Background and Aim:**

Parasitic infection commonly affects freshwater ornamental fishes. Parasites in fish may impede their growth and even cause death, resulting in a decline in fecundity. The prevalence of lernaeosis in aquaculture ponds in Indonesia requires attention because of missing data, especially from Yogyakarta. Therefore, this study aimed to identify the *Lernaea* species found in fish in Indonesia, particularly in Yogyakarta, molecularly and morphologically, as well as an overview of their distribution and the water condition they inhabit.

**Materials and Methods:**

*Lernaea* species were collected from three different fish species in two districts of Yogyakarta, Indonesia, for precise identification. *Lernaea* specimens were characterized morphologically and subjected to molecular identification based on *18S rRNA* and *28S rRNA* genes.

**Results:**

*Lernaea* in this study was morphologically and genetically confirmed as *Lernaea cyprinacea*, and the infection rate in each fish species was different. Water conditions might have contributed to the differences in infection levels.

**Conclusion:**

This study characterized *L. cyprinacea* isolated from Yogyakarta. Future research should focus on sequencing as much molecular information as possible and carrying out more experimental infections.

## Introduction

Infectious disease outbreaks in fish farming are the most important issue that farmers must manage to avoid losses [1]. Due to an imbalance between the environment, host, and pathogenic agents, illnesses may arise in aquatic organisms, including fish. Stress may be caused by improper aquaculture management, making fish susceptible to infection and immunocompromised [2, 3]. Parasitic infection is a common illness affecting freshwater ornamental fishes. Parasites in fish may impede development and cause death, resulting in decreased fecundity [4].

Lernaeosis is a fish illness caused by Copepod ectoparasites of the genus *Lernaea* [5]. These ectoparasites attach to all exterior parts of the fish, including some internal parts such as the mouth, gills [6, 7], gill filaments, or even the eyes [8, 9]. *Lernaea* are usually associated with significant mortality in aquaculture, and the effect is quite severe, as fish death may occur in massive numbers. Due to the widespread introduction of tropical fishes such as cyprinids, *Lernaea* *cyprinacea* has become the most widespread lernaeid [10, 11]. At present, the holdfast of metamorphosed females is used to identify *Lernaea* species. Several studies have shown that the shape of holdfast alters significantly due to lernaeosis [5]. Consequently, more accurate approaches have been developed for identification using DNA sequencing technologies targeting the *18S rRNA*, *28S rRNA*, and cytochrome c oxidase subunit 1 gene regions [11, 12].

The occurrence of lernaeosis in aquaculture ponds in Indonesia requires attention because it causes a reduction in fish production and quality [9, 13]. This study aimed to obtain information on *Lernaea* species infecting fish in Indonesia, particularly in Yogyakarta. The morphological and molecular identification studies were undertaken to determine the taxonomic status of this parasite in the region and provide an overview of their range and interaction with the water conditions in which the fish is raised.

## Materials and Methods

### Ethical approval

This study was approved by the Ethical Clearance Committee team of the Faculty of Veterinary Medicine, Universitas Gadjah Mada (letter number 0074/EC-FKH/Int./2019), and the experiments were conducted following the ethical principles of the use and treatment of fish.

### Study period and location

The study was conducted from January 2020 to February 2021. The samples were collected from two districts, namely Sleman and Bantul, in Yogyakarta Province, Indonesia. The samples were processed at the Laboratory of Parasitology, Faculty of Veterinary Medicine, Universitas Gadjah Mada.

### Sampling sites

Fish samples consisting of koi (*Cyprinus carpio*), comet (*Carassius auratus*), and goldfish (*C. auratus*) were collected from three different sites across the two districts. The sites from Sleman, Yogyakarta, were located at 7° 41’ 21’ latitude and 110° 21 4” longitude, with 10 samples at 7° 48’ 42” latitude and 110° 28’ 57” (n = 30), and 7° 43’ 46” latitude and 110° 24’ 28” longitude (n = 20). The location from Bantul, Yogyakarta, 7° 47’ 6’ latitude and 110° 16’ 43” longitude (n = 22), 7° 49’ 4” latitude and 110° 19’ 55” (n = 30), and 7° 50’ 57” latitude and 110° 22’ 53” longitude (n = 25). The samples were placed in an oxygenated plastic bag and sent to the Parasitology Laboratory of the Faculty of Veterinary Medicine at the Universitas Gadjah Mada.

Fish samples were tallied to determine whether they were positive or negative for *Lernaea* infection. The number of *Lernaea* on each fish was counted in positive samples to evaluate the degree of infestation. According to Kriswijayanti *et al*. [13], the degree of *Lernaea* infestation was light if 1–5 *Lernaea* were detected on one fish, moderate if 6–10 *Lernaea* were found on one fish, and heavy if infected with more than 10 *Lernaea*.

### Morphological identification

*Lernaea* were taken from the fish body using tweezers, rinsed with physiological NaCl, preserved with lactophenol, and mounted in Canada balsam for morphological identification [14]. The Lernaea samples used for molecular identification were preserved in absolute ethanol. *Lernaea* were observed in the holdfast, abdomen, position, and number of legs. The body and anchor length were measured. *Lernaea* were identified using Kabata’s [15] identification key.

### Water quality analysis

Pond water quality testing was conducted by an expert at the Yogyakarta Health Laboratory by collecting one liter of water samples and fish. The water quality was determined by measuring the biochemical oxygen demand (BOD), chemical oxygen demand (COD), hydrogen sulfide, nitrite (NO_2_), nitrate (NO_3_), free ammonia (NH_3_), and pH. Water samples were collected between November 2020 and January 2021 and water quality testing was performed on the same day.

### Molecular analysis

The genomic DNA of the parasite was extracted according to the manufacturer’s instructions using a GeneJET Genomic DNA Purification Kit (Thermo Scientific^®^, Vilnus, Lithuania). The following primers from Song *et al*. [12] were used: 18SF (5’-AAG GTG TGM CCT ATC AAC T-3’), 18SR (5’-TTA CTT CCT CTA AAC GCT C-3’), 28SF (5’-ACA ACT GTG ATG CCC TTA G-3’), and 28SR (5’-TGG TCC GTG TTT CAA GAC G-3’). Cycle settings for amplified 18S regions were 94°C for 5 min, followed by 30 cycles of 94°C for 30 s, 54°C for 30 s, and 72°C for 1 min, with a final extension at 72°C for 5 min [12]. The conditions for the polymerase chain reaction (PCR) reactions targeting the 28S regions and PCR cycling parameters were according to Pallavi *et al*. [16]. The mixtures for PCR reaction containing 2 μL genomic DNA, 0.6 μL (0.2 μMol) of each forward primer and reverse primer, 6.8 microliters of ddH_2_O, and ten microliters of 2 PCR GoTaq^®^ Green Master Mix (Madison, Wisconsin) were combined in total 20 μL volume. The amplified fragments were visualized on 0.8% agarose gel, and electrophoresis was performed at 100 V for 30 min.

### DNA sequencing and phylogenetic analysis

Specific amplified products were delivered to the Genetika Science Company, in Banten, Indonesia, for automated sequencing. The sequences were manually reviewed and adjusted for correctness using BioEdit Software version 7.2.5 [17], freely accessed at www.mbio.ncsu.edu. Other *Lernaea* and Lernaeidae sequences were acquired from GenBank and aligned using CrustalW in MEGA version 10.2.6 (https://www.megasoftware.net/).

The Maximum-likelihood method was used to construct phylogenetic trees of 18S and *28S rRNA* sequences from *Lernaea* isolates. The bootstrap value (1000 repetitions) was used to determine the proportion of duplicate trees in which the linked taxa were grouped. The Kimura 2-parameter approach was used to determine evolutionary distances [16].

### Statistical analysis

A correlation test was used to examine the association between the degree of infestation and the water quality in each pond. The p-value for each water quality measurement group was determined using GraphPad Prism 8 (https://www.graphpad.com/).

## Results

### *Lernaea* identification

**Figure-1:**
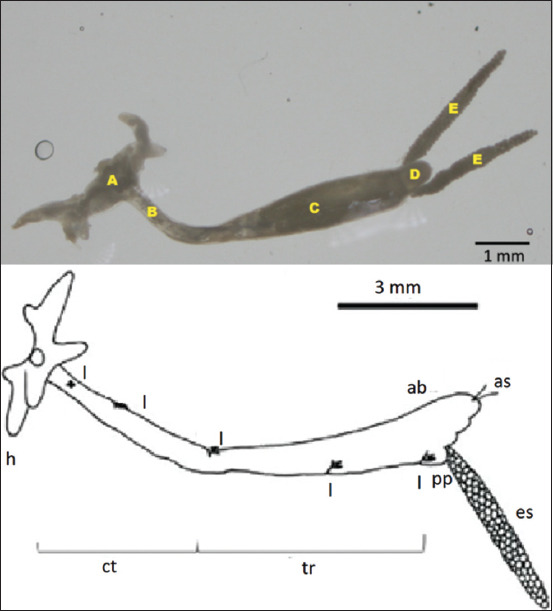
Comparison of holdfast morphology in *Lernaea* *cyprinacea* found with literature. *L. cyprinacea* found in the study (left). (A) Holdfast, (B) cephalothorax, (C) trunk, (D) abdomen, and (E) egg sacs. Schematic morphology of *L. cyprinacea* (right): ab=Abdomen, as=Anal setae, ct=Cephalothorax, es=Egg sac, h=Head, pp=Pregenital prominence, l=Legs, tr=Trunk [21].

*Lernaea* were acquired from two fish species in the *Cyprinidae* family: Koi (*C. carpio*), comet fish (*C. auratus*), and goldfish (*C. auratus*). Each fish variety had different infection rates (Table-1). The prevalence of the parasites was calculated as a percentage by dividing the number of fish infected by the number of fish observed [18, 19]. The number of *Lernaea* identified in each fish ranged between 1 and 7, and the amount of infection observed in this investigation was graded as low, except for comet fish from Bantul, which was graded as moderate. Based on their morphology, the *Lernaea* discovered belonged to *L. cyprinacea*. The parasite was found on the scales and tail fins of fish, ranging from 10 mm to 13 mm. *Lernaea*’s morphology revealed a tiny, semi-spherical cephalothorax linked to the first swimming limb in the middle of the holdfast system. The second and fourth leg segments were in the neck and abdomen, respectively. The body is not segmented. The holdfast is divided into two branches: Dorsal and ventral. Ventral branches appeared thinner and unbranched, whereas dorsal branches contained two further branches. A 1–2 mm long egg sac was found in the posterior region of female *L*. *cyprinacea*. The morphology concords with the description of *L. cyprinacea* in the previous studies [15, 20], which specifies that *L. cyprinacea* has a holdfast that branches into two pairs. While the small holdfast is unbranched and appears shorter, a larger T-shaped holdfast is installed. According to Kabata [15], the total length of *L. cyprinacea* ranges between 10 and 20 mm. Figure-1 [21] depicts the morphological similarities between *L. cyprinacea* discovered in this study and those reported in the literature.

**Table-1:** Number of infected fish and water condition.

Location	Fish	Total no. of fish examined	NI	Prevalence (%)	Parasite/fish (max)	Water condition						

						Temp. (°C)	pH	DO (mg/L)	NO_2_ (mg/L)	NO_3_ (mg/L)	NH_3_ (mg/L)	BOD
Sleman	Koi	10	6	60	3	25	8.3	6.36	0.48	2.92	0.04	<0.86
	Comet	30	12	40	5	27	7.7	5.88	1.34	3.70	0.03	1.00
	Goldfish	20	8	40	5	25	8.0	5.75	2.71	16.06	0.06	<0.86
Bantul	Koi	22	6	27	4	25	6.6	8.67	2.74	11.52	0.03	1.04
	Comet	30	5	16	7	28	7.3	7.13	0.60	16.00	0.04	<0.86
	Goldfish	25	3	12	2	26	6.8	6.86	0.93	13.50	0.05	<0.86
p-value						0.17*	0.01	0.17*	0.49*	0.05*	0.34*	0.49*

NI=Number infected,

* Not significant or *P>*0.05, BOD=Biochemical oxygen demand, DO: Dissolved oxygen, NO_2_=Nitrite, NO_3_=Nitrate, NH_3_=Free ammonia

### Water quality analysis

Water quality was determined based on temperature, pH, dissolved oxygen (DO), NO_2_, NO_3_, and ammonia levels. The data reveal that the temperature of the water pond in this research varied between 25 and 28.5°C, with a pH of 6.67–8.30, DO of 5.75–8.67 mg/L, NO_2_ of 0.487–2.748 mg/L, NO_3_ of 2.92–16.06 mg/L, and ammonia of 0.035–0.06 mg/L. These data show that the water quality in the fishpond was still within the normal range and could be tolerated by the fish and parasites. The correlation analysis between the prevalence of *L. cyprinacea* and indicators of water condition showed that only pH was a significant factor (p = 0.01) that influenced the infestation of *L. cyprinacea*.

### Molecular analysis

**Figure-2:**
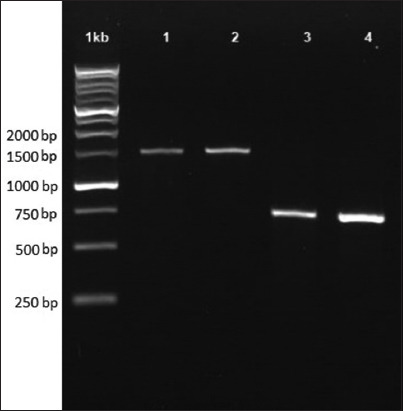
Picture of an agarose gel with polymerase chain reaction results from the 28S ribosomal RNA (*18S rRNA*) and *28S rRNA* region of isolated *Lernaea* parasites on *Carassius auratus*. (1) *18S rRNA* gene from Sleman isolate, (2) *18S rRNA* gene from Bantul isolate, (3) *28S rRNA* from Sleman isolate, and (4) *28S rRNA* from Bantul isolate.

We successfully amplified a 1332 bp *Lernaea* isolate using the *18S rRNA* region by PCR, whereas the *28S rRNA* amplified a 715 bp product (Figure-2). After the manual removal of *18s rRNA* using BioEdit, the low-quality chromatogram was deleted. Unfortunately, only one of the two *18S rRNA* sequences showed a good chromatogram. Therefore, we used only one *18S rRNA* sequence for phylogenetic analysis. The low-quality chromatogram of *28s rRNA* was manually trimmed and removed. The 18S and *28S rRNA* sequences from the *Lernaea* isolates were deposited in GenBank under accession numbers OP895710, OP902215, and OP902216, respectively. All sequences retrieved in this study were genetically similar to *L*. *cyprinacea*, as determined by National Center for Biotechnology Information nucleotide-BLAST (USA) (https://blast.ncbi.nlm.nih.gov/Blast.cgi) analysis [12].

**Figure-3:**
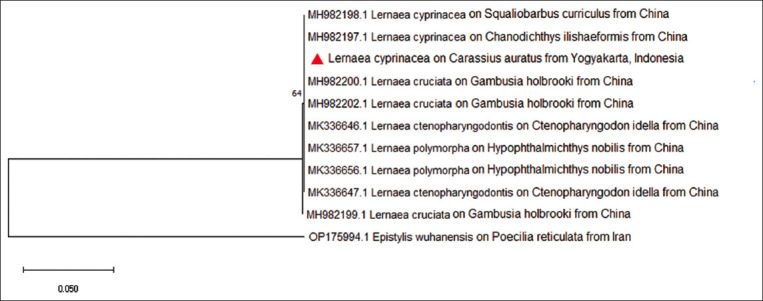
Phylogenetic tree generated by maximum-likehood method based on the *18S rRNA*. *Lernaea* sequences obtained in this study were grouped to *Lernaea cyprinacea* from several countries.

**Figure-4:**
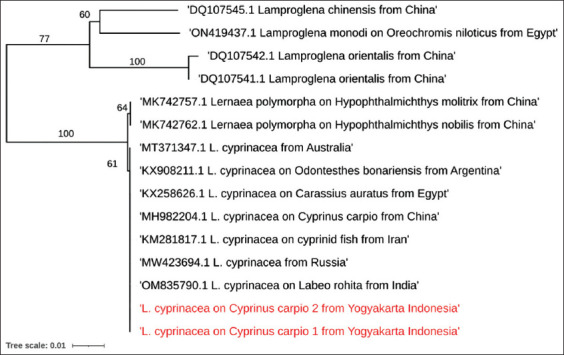
Maximum-likelihood phylogenetic tree constructed using the *28S rRNA*. Lernaea sequences in this study were combined to *Lernaea cyprinacea* from different nations.

The maximum-likelihood phylogeny of the *18S rRNA* gene region samples collected in this study clustered into a single clade with other *L. cyprinacea* collected from different species in many countries (Figure-3). High levels of genetic similarity were found in the sequence of the *28S rRNA* for *L. cyprinacea* from Yogyakarta and those from other geographic origins (Figure-4), including India (OM835790), Russia (MW423694), Australia (MT371347.1), China (MH982204.1), and Iran (KM281817.1) with 100% identity and also 100% identic to the isolate from Egypt (KX258626.1).

## Discussion

Indonesia has a very wide ornamental fish development potential, with 400 out of 1100 species of freshwater ornamental fish marketed internationally; however, only 90 species can be maintained by the public. Most ornamental fish cultivation in Yogyakarta occurs in the Sleman and Bantul districts, where there are suitable areas for fish ponds [22, 23]. Fish ectoparasites are among the most significant issues related to pond fish culture. Consequently, several parasites are destructive to farmed fish and have become a species of interest [24]. Morphological identification of *Lernaea* that infects koi, comet, and goldfish in the Bantul and Sleman areas revealed only one species, *L. cyprinacea*. The parasite is known to infect a variety of freshwater fish, primarily those of the *Cyprinidae* family [25]. This species has been reported in 30 fish species, including *C. auratus* and *C. carpio*, discussed in this study [26]. *Lernaea cyprinacea* was discovered on the ventral, anal, and caudal fins of several *Poecilia reticulata* species in a study conducted in the Kingdom of Saudi Arabia [27]. At the attachment site, which appeared red and ulcerated, intense focal inflammation and hemorrhage were readily visible. The total prevalence of infection was 68.1% (32/47). Infection was more prevalent in females (29/38; 76.3%) than in males (3/9; 33.3%) [27]. The *Lernaea* species discovered in koi, comet, and goldfish in this study were *L. cyprinacea*. In addition to infecting fish of the *Cyprinidae* family, *L. cyprinacea* has been documented to infect *Scleropages jardinii* in Indonesia [28]. *Lernaea* *cyprinacea* can be found in freshwater in Argentina [10], Iraq [26], Kingdom of Saudi Arabia [27], Indonesia [28], Europe [29], Africa [30], India [31], Pakistan [32], and New Mexico and Texas [33]. South-east Asia, especially Indonesia, offers a tropical environment where this parasite may thrive. The average body length and anchor width of *L. cyprinacea* parasites obtained from experimental infections were 8.86 ± 1.71 and 2.31 ± 1.41 mm, respectively [5].

The parameters to assess water quality in the study were water temperature, pH, DO, NO_2_, NO_3_, NH_3_, BOD, and COD. These eight factors interact with one another and affect the survival of fish and parasites. Water temperature influences fish metabolism and immunity; the lower the temperature, the more sensitive it is to illness [9, 34]. *Lernaea* thrives between 26 and 28°C. If temperatures drop below 20°C, juvenile *Lernaea* cannot complete their development, and at 14°C, females are incapable of reproduction [34]. However, adult females are able to overwinter their fish hosts and produce eggs when water temperatures rise in spring [35]. The previous studies conducted by Raissy *et al*. [36] and Stavrescu-Bedivan *et al*. [37] found that increased water temperature could affect the prevalence of *L. cyprinacea*. Freshwater fish prefer a slightly acidic pH range (6.5–6.8 with a pH tolerance range of 6.5–9.0, and this study showed that a higher alkaline pH might increase infestation of *L. cyprinacea*.

The most essential element that influences fish health is the amount of oxygen in water. Temperature and water salinity considerably affect DO levels; the greater the temperature and salinity of the water, the lower the DO levels [2]. Freshwater quality factors in this range marginally restrict *Lernaea* growth and reproduction. The findings of the study revealed the quality of pond water that fish could still accept. This situation is consistent with parasite proliferation. Therefore, *Lernaea* may still be observed on the fish body, but with a decreased degree of infection in certain areas. Water conditions may have affected the heterogeneity in the degree of infection. The pH, oxygen, temperature, and salinity values were within the anticipated range [38, 39]. Along with stocking density, climatic factors, and environmental conditions play a significant role in the prevalence of Lernaeid ectoparasites at various geographic locations [4].

The *28S rRNA* sequences of *L. cyprinacea* from Yogyakarta and those from other geographic origins showed a high genetic similarity. The sequences indicated that the species *L. cyprinacea* based on the *28S rRNA* gene sequences showed 100% identity between the specimens, regardless of where they were found geographically. According to the BLAST result of *18S rRNA* and *28S rRNA*, we found differences from the same sample. The *18S rRNA* gene was similar to *L. cruciata* (MH982203.1) with homology 100%, while the *28S rRNA* gene is similar to *L. cyprinacea*. (OM835790.1) with homology 100%. A more thorough technique based on molecular data is necessary to continue characterizing *Lernaea* species. According to Kabata [15], there are approximately 37 distinct *Lernaea* species. Since molecular biology has emerged as a valuable integrative tool for morphological identification, the number of valid *Lernaea* species is projected to decline. According to Hua *et al*. [5], the molecular profiles of *L. cyprinace*a and *L. cruciat*a may be synonymous. The phylogenetic tree of *18S rRNA* in our study also showed that *L. cyprinacea* and *L. cruciata* were in the same clade. There might be limited resources available on GenBank regarding *Lernaea*. Hence, the anchor form cannot be relied upon to distinguish between *Lernaea* species because of host-induced morphological diversity. Despite the advancements in molecular ecology and population genetics of this parasite in freshwater aquaculture, many traits are yet to be adequately explored.

## Conclusion

*Lernaea* species in this study were morphologically and genetically identified as *L. cyprinacea*. This study indicated that pond water quality could tolerate *Lernaea* infestation and the conditions were favorable for parasite multiplication. Thus, *Lernaea* may still be visible on the body surface of fish, but with a reduced degree of infection in particular regions.

## Authors’ Contributions

JP, DP, AS, and YRN: Designed the study and contributed to sample collection. AS, VIN, and WN: Analysis and interpretation of the data. JP, YRN, WN, and VIN: Drafted and revised the manuscript. All authors have read, revised, and approved the manuscript.

## References

[1] Piasecki W, Goodwin A.E, Eiras J.C, Nowak B.F (2004). Importance of *Copepoda* in freshwater aquaculture. Zoo. Stud..

[2] Noga E.J (2010). Fish Disease:Diagnosis and Treatment.

[3] de Freitas Souza C.D.F, Baldissera M.D, Baldisserotto B, Heinzmann B.M, Martos-Sitcha J.A, Mancera J.M (2019). Essential oils as stress-reducing agents for fish aquaculture:A review. Front. Physiol..

[4] Bilal M, Abbas F, Atique U, Rehman M.H, Inayat M, Zohaib M, Saleem M, Fatima S, Sherazi S.W.S, Tehreem A, Ali A, Sanwal M.U, Abdullah M, Ullah M, Mubeen N (2024). Lernaeid parasites prevalence in commercial freshwater fish species at various fish farms in Pakistan. Braz. J. Biol..

[5] Hua C.J, Zhang D, Zou H, Li M, Jakovlić I, Wu S.G, Wang G.T, Li W.X (2019). Morphology is not a reliable taxonomic tool for the genus *Lernaea*:Molecular data and experimental infection reveal that *L. cyprinacea* and *L. cruciata* are conspecific. Parasit. Vectors.

[6] Noga E.J (1986). The importance of *Lernaea cruciata* (Le Sueur) in the initiation of skin lesions in largemouth bass, *Micropterus salmoides* (Lacepede), in the Chowan River, North Carolina, USA. J. Fish Dis..

[7] Barson M, Mulonga A, Nhiwatiwa T (2008). Investigation of a parasitic outbreak of *Lernaea cyprinacea* Linnaeus (*Crustacea*:*Copepoda*) in fish from Zimbabwe. Afr. Zool..

[8] Woo P, Shariff M (2006). *Lernea cyprinacea* L. (*Copepoda*:Caligidea) in *Helostoma temmincki* Cuvier and Valenciennes:The dynamics of resistance in recovered and naive fish. J. Fish Dis.

[9] Nur F.M, Batubara A.S, Fadli N, Rizal S, Siti-Azizah M.N, Wilkes M, Muchlisin Z.A (2022). *Lernaea cyprinacea* Linnaeus, 1758 (*Copepoda*:Lernaeidae) infection on Betta Rubra Perugia, 1893 (*Anabantiformes*:*Osphronemidae*) from Aceh Province, Indonesia. Rev. Bras. Parasitol. Vet..

[10] Soares I.A, Salinas V, del Ponti O, Mancini M.A, Luque J.L (2018). First molecular data for *Lernaea cyprinacea* (*Copepoda*:*Cyclopoida*) infesting *Odontesthes bonariensis*, a commercially important freshwater fish in Argentina. Rev. Bras. Parasitol. Vet..

[11] Zhu X, Barton D.P, Wassens S, Shamsi S (2021). Morphological and genetic characterisation of the introduced copepod *Lernaea cyprinacea* Linnaeus (*Cyclopoida*:Lernaeidae) occurring in the Murrumbidgee catchment, Australia. Mar. Freshw. Res..

[12] Song Y, Wang G.T, Yao W.J, Gao Q, Nie P (2008). Phylogeny of freshwater parasitic copepods in the Ergasilidae (*Copepoda*:*Poecilostomatoida*) based on 18S and 28S rDNA sequences. Parasitol. Res..

[13] Kriswijayanti B.D, Kismiyati K, Kusnoto K (2019). Identifikasi dan derajat infestasi *Lernaea* pada ikan maskoki *(Carassius auratu*s) di kabupaten Tulungagung, Jawa Timur (Identification and Degrees of *Lernaea* Infestation in Goldfish *(Carassius auratu*s) at Tulungagung, East Java). J. Aquac. Fish Health.

[14] Fernando C.H (1972). Methods for the Study of Freshwater Fish Parasites. University of Waterloo Biology Series. Department of Biology, University of Waterloo, Canada.

[15] Kabata Z (1985). Parasites and Diseases of Fish Culture in the Tropics. Taylor and Francis, London.

[16] Pallavi B, Shankar K.M, Abhiman P.B, Ahmed I (2017). Molecular identification of the fish parasite *Lernaea*. Indian J. Fish..

[17] Hall T.A (1999). BioEdit:A user-friendly biological sequence alignment editor and analysis program for Windows 95/98/NT. Nucleic Acids Symp. Ser..

[18] Raja R.A, Patil P.K, Avunje S, Kumaran M, Solanki H.G, Jithendran K.P, Alavandi S.V, Vijayan K.K (2022). Efficacy of emamectin benzoate in controlling natural infestations of ectoparasites in economically important fish species of India. Aquaculture.

[19] Raja R.A, Patil P.K, Avunje S, Aravind R.P, Alavandi S.V, Vijayan K.K (2020). Biosafety, withdrawal and efficacy of anti-parasitic drug emamectin benzoate in Asian Seabass (*Lates calcarifer*). Aquaculture.

[20] Demaree R.S (1967). Ecology and external morphology of *Lernaea cyprinacea*. Am. Mid. Nat..

[21] Gervasoni S.H, Chemes S.B, Scaglione M, Cerutti R.D (2018). First report of *Lernaea cyprinacea* (*Crustacea*:Lernaeidae) parasitising *Rhamdia quelen* (Pisces:Heptapteridae) in Santa Fe (Argentina) under hatchery conditions. Rev. Colomb. Cienc. Pecu..

[22] Mulya M.A, Darmawangsa G.M, Wali R.M, Santoso S.J.J (2021). Pembenihan ikan koi *Cyprinus rubrofuscu*s (Lacepede, 1803) di mina karya koi, kabupaten Sleman, Daerah Istimewa Yogyakarta (Hatchery and Intermediate Rearing of Koi Carp *Cyprinus rubrofuscu*s (Lacepede, 1803) at Mina Karya Koi, Sleman Regency, Special Region of Yogyakarta). J. Sains Ter.

[23] Iskandar A, Amalia D, Aji H.S, Hendriana A, Darmawangsa G.M (2021). Optimalisasi pembenihan ikan koi *Cyprinus rubrofuscus* di Mina Karya Koi, Sleman, Yogyakarta (Breeding optimization on Koi Carp *Cyprinus rubrofuscu*s at Mina Karya Koi, Sleman Regency, Yogyakarta). J. Fish. Mar. Sci.

[24] Tufail H, Qureshi N.A, Khan N, Iqbal K.J, Khan M.R, Maqbool A (2017). Prevalence and effects of *Lernaea cyprinacea* (anchor worm) on the growth, skin histopathology and hematology of *Catla catla*. Iran. J. Fish. Sci..

[25] Lester R.J.G, Hayward C.J, Woo T.K (2006). Phylum Arthropoda. Fish diseases and disorders. Volume 1:Protozoan and metazoan infections.

[26] Mhaisen F (2021). Checklist of fish hosts of species of *Lernaea Linnaeus*, 1758 (*Hexanauplia*:*Cyclopoida*:Lernaeidae) in Iraq. Bio. App. Environ. Res..

[27] Ghobashy M, Shafeey H.A, Taeleb A (2018). Guppy (*Poecilia*) *Poeciliidae* fish naturally infected with *Lernaea cyprinacea* parasites (Linnaeus 1758) in KSA. Parasitol. United J..

[28] Shatrie D.N, Imamudin K, Nurcahyo W, Triyanto T (2011). Identification of *Lernaea* spp. which infected Arwana irian fish {*Scleropages jardinii* (Saville-kent, 1892)}in Merauke, Jakarta, Bogor, and Depok. Berita Biol.

[29] Ahnelt H, Konecny R, Gabriel A, Bauer A, Pompei L, Lorenzoni M, Sattmann H (2018). First report of the parasitic copepod *Lernaea cyprinacea (Copepoda*:*Lernaeidae*) on gobioid fishes (*Teleostei*:Gobonellidae) in southern Europe. Knowl. Manag. Aquat. Ecosyst..

[30] Avenant-Oldewage A, Robinson J (1996). Aspects of the morphology of the parasitic copepod *Lernaea cyprinacea* Linnaeus, 1758 and notes on its distribution in Africa. J. Crustac..

[31] Shivaji C, Vaishali L, Suduwar M, Kannewad P (2016). Larnaea cruciata (*Crustacea*:*Copepoda*) first report on infection to *Notopterus kapirat* in the Godavari River, Marathwada region, India. Int. J. Curr. Res. Acad. Rev..

[32] Iqbal Z, Shafqat A, Haroon F (2012). *Lernaea* diversity and infection in Indian and Chinese carps under semi-intensive culture conditions in Lahore, Punjab. J. Anim. Plant Sci.

[33] Durham B.W, Bonner T.H, Wilde G.R (2002). Occurrence of *Lernaea cyprinacea* on Arkansas River shiners and peppered chubs in the Canadian River, New Mexico and Texas. Southwest. Nat..

[34] Hossain M.M.M, Ferdoushi J, Rupom A.H (2018). Biology of anchor worms (*Lernaea cyprinacea*). J. Entomol. Zool. Stud..

[35] Steckler N, Yanong R.P (2013). *Lernaea* (Anchorworm) infestations in Fish:FA185, 12/2012. EDIS.

[36] Raissy M, Sohrabi H, Rashedi M, Ansari M (2013). Investigation of a parasitic outbreak of *Lernaea cyprinacea* Linnaeus (*Crustacea*:*Copepoda*) in Cyprinid fish from Choghakhor lagoon. Iran. J. Fish. Sci..

[37] Stavrescu-Bedivan M.M, Popa O.P, Popa L.O (2014). Infestation of *Lernaea cyprinacea (Copepoda*:Lernaeidae) in two invasive fish species in Romania, *Lepomis gibbosus* and *Pseudorasbora parva*. Knowl. Manag. Aquat. Ecosyst..

[38] Mancini M, Guagliardo S, Del Ponti O, Salinas V, Regis L, Tanzola D (2021). Ecology and implications of parasitism by *Lernaea cyprinacea* (*Crustacea*:*Copepoda*) on Argentinian silverside *Odontesthes bonariensis* (*Teleostei*:*Atherinopsidae*). Pan-Am. J. Aquat. Sci..

[39] İnnal D (2020). Detection of ectoparasite *Lernaea cyprinacea (Copepoda*:Lernaeidae) on some cypriniformes fish from the Mediterranean region of Turkey. Commagene J. Biol..

